# Meta-Analysis Identifies Gene-by-Environment Interactions as Demonstrated in a Study of 4,965 Mice

**DOI:** 10.1371/journal.pgen.1004022

**Published:** 2014-01-09

**Authors:** Eun Yong Kang, Buhm Han, Nicholas Furlotte, Jong Wha J. Joo, Diana Shih, Richard C. Davis, Aldons J. Lusis, Eleazar Eskin

**Affiliations:** 1 Department of Computer Science, University of California, Los Angeles, Los Angeles, California, United States of America; 2 Division of Genetics, Brigham & Women's Hospital, Harvard Medical School, Boston, Massachusetts, United States of America; 3 Partners HealthCare Center for Personalized Genetic Medicine, Boston, Massachusetts, United States of America; 4 Program in Medical and Population Genetics, Broad Institute of Harvard and MIT, Cambridge, Massachusetts, United States of America; 5 Interdepartmental Program in Bioinformatics, University of California, Los Angeles, Los Angeles, California, United States of America; 6 Department of Medicine, University of California, Los Angeles, Los Angeles, California, United States of America; 7 Department of Human Genetics, University of California, Los Angeles, Los Angeles, California, United States of America; Georgia Institute of Technology, United States of America

## Abstract

Identifying environmentally-specific genetic effects is a key challenge in understanding the structure of complex traits. Model organisms play a crucial role in the identification of such gene-by-environment interactions, as a result of the unique ability to observe genetically similar individuals across multiple distinct environments. Many model organism studies examine the same traits but under varying environmental conditions. For example, knock-out or diet-controlled studies are often used to examine cholesterol in mice. These studies, when examined in aggregate, provide an opportunity to identify genomic loci exhibiting environmentally-dependent effects. However, the straightforward application of traditional methodologies to aggregate separate studies suffers from several problems. First, environmental conditions are often variable and do not fit the standard univariate model for interactions. Additionally, applying a multivariate model results in increased degrees of freedom and low statistical power. In this paper, we jointly analyze multiple studies with varying environmental conditions using a meta-analytic approach based on a random effects model to identify loci involved in gene-by-environment interactions. Our approach is motivated by the observation that methods for discovering gene-by-environment interactions are closely related to random effects models for meta-analysis. We show that interactions can be interpreted as heterogeneity and can be detected without utilizing the traditional uni- or multi-variate approaches for discovery of gene-by-environment interactions. We apply our new method to combine 17 mouse studies containing in aggregate 4,965 distinct animals. We identify 26 significant loci involved in High-density lipoprotein (HDL) cholesterol, many of which are consistent with previous findings. Several of these loci show significant evidence of involvement in gene-by-environment interactions. An additional advantage of our meta-analysis approach is that our combined study has significantly higher power and improved resolution compared to any single study thus explaining the large number of loci discovered in the combined study.

## Author Summary

Identifying gene-by-environment interactions is important for understand the architecture of a complex trait. Discovering gene-by-environment interaction requires the observation of the same phenotype in individuals under different environments. Model organism studies are often conducted under different environments. These studies provide an unprecedented opportunity for researchers to identify the gene-by-environment interactions. A difference in the effect size of a genetic variant between two studies conducted in different environments may suggest the presence of a gene-by-environment interaction. In this paper, we propose to employ a random-effect-based meta-analysis approach to identify gene-by-environment interaction, which assumes different or heterogeneous effect sizes between studies. Our approach is motivated by the observation that methods for discovering gene-by-environment interactions are closely related to random effects models for meta-analysis. We show that interactions can be interpreted as heterogeneity and can be detected without utilizing the traditional approaches for discovery of gene-by-environment interactions, which treats the gene-by-environment interactions as covariates in the analysis. We provide a intuitive way to visualize the results of the meta-analysis at a locus which allows us to obtain the biological insights of gene-by-environment interactions. We demonstrate our method by searching for gene-by-environment interactions by combining 17 mouse genetic studies totaling 4,965 distinct animals.

## Introduction

Identifying environmentally specific genetic effects is a key challenge in understanding the structure of complex traits. In humans, gene-by-environment (GxE) interactions have been widely discussed [1]–[12] yet only a few have been replicated. One reason for this discrepancy is the inability to accurately control for environmental conditions in humans as well as the inability to observe the same individuals in multiple distinct environments. Model organisms do not share such difficulties and for this reason can play a crucial role in the identification of gene-by-environment interactions. For example, in many mouse genetic studies the same traits are examined under different environmental conditions. Specifically, knock-out or diet-controlled mice are often utilized in the study of cholesterol levels. The availability of these studies presents a unique opportunity to identify genomic loci involved in gene-by-environment interactions as well as those loci involved in the trait independent of the environment.

In order to utilize genetic studies in model organisms to identify gene-by-environment interactions, one needs to directly compare the effects of genetic variations in studies conducted under different conditions. This practice is complicated for a number of reasons, when combining more than two studies. First, environmental conditions are often variable across studies and do not fit to the standard univariate model for interactions. For example, in one study, cholesterol may be examined under different diet conditions (eg. low fat and high fat) and then in another study cholesterol is examined using gene knockouts. In this case, it is not straightforward to analyze these studies in aggregate using a single variable to represent the environmental condition. Applying a multivariate model, one in which the environment is represented using multiple environmental variables, results in increased degrees of freedom and low statistical power. Second, model organisms such as the mouse exhibit a large degree of population structure. Population structure is well-known for causing false positives and spurious associations [13], [14] in association analysis and can be expected to complicate the ability to combine separate studies.

In this paper, we propose a random-effects based meta-analytic approach to combine multiple studies conducted under varying environmental conditions and show that this approach can be used to identify both genomic loci involved in gene-by-environment interactions as well as those loci involved in the trait independent of the environment. By making the connection between gene-by-environment interactions and random effects model meta-analysis, we show that interactions can be interpreted as heterogeneity and detected without requiring uni- or multi-variate models. We also define an approach for correcting population structure in the random effects model meta-analysis, extending the methods developed for fixed effects model meta-analysis [15]. We show that this method enables the analyses of large scale meta-analyses with dozens of heterogeneous studies and leads to dramatic increases in power. We demonstrate that insights regarding gene-by-environment interactions are obtained by examining the differences in effect sizes among studies facilitated by the recently developed m-value statistic [16], which allows us to distinguish between studies having an effect and studies not having an effect at a given locus.

We applied our approach, which we refer to as Meta-GxE, to combine 17 mouse High-density lipoprotein (HDL) studies containing 4,965 distinct animals. To our knowledge, this is the largest mouse genome-wide association study conducted to date. The environmental factors of the 17 studies vary greatly and include various diet conditions, knock-outs, different ages and mutant animals. By applying our method, we have identified 26 significant loci. Consistent with the experience of meta-analysis in human studies, our combined study finds many loci which were not discovered in any of the individual studies. Among the 26, 24 loci have been previously implicated in having an effect on HDL cholesterol or closely related lipid levels in the blood, while 2 loci are novel findings. In addition, our study provides insights into genetic effects on several disease loci and their relationship between environment and sex. For example, we identified 3 loci (Chr10:21399819, Chr19:3319089, ChrX:151384614), where female mice show a more significant effect on HDL phenotypes than male mice. We also identified 7 loci (Chr1:171199523, Chr8:46903188, Chr8:64150094, Chr8:84073148, Chr10:90146088, Chr11:69906552, Chr15:21194226) where male mice show a more significant effect on HDL than female mice. In addition, many of the loci show strong gene-by-environment interactions. Using additional information describing the studies and our predictions of which studies do and do not contain an effect, we gain insights into the interaction. For example, locus on chromosome 8 (Chr8:84073148) shows a strong sex by mutation-driven LDL level interaction, which affects HDL cholesterol levels.

Part of the reason for our success in identifying a large number of loci is that our study combined multiple mouse genetic studies many of which use very different mapping strategies. Over the past few years, many new strategies have been proposed beyond the traditional F2 cross [17] which include the hybrid mouse diversity panel (HMDP) [18], [19], heterogeneous outbred stocks [20], commercially available outbred mice [21], and the collaborative cross [22]. In our current study, we are combining several HMDP studies with several F2 cross studies and benefit from the statistical power and resolution advantages of this combination [15]. The methodology presented here can serve as a roadmap for both performing and planning large scale meta-analysis combining the advantages of many different mapping strategies. Meta-GxE is publicly available at http://genetics.cs.ucla.edu/metagxe/.

## Results

### Discovering environmentally-specific loci using meta-analysis

The Meta-GxE strategy uses a meta-analytic approach to identify gene-by-environment interactions by combining studies that collect the same phenotype under different conditions. Our method consists of four steps. First, we apply a random effects model meta-analysis (RE) to identify loci associated with a trait considering all of the studies together. The RE method explicitly models the fact that loci may have different effects in different studies due to gene-by-environment interactions. Second, we apply a heterogeneity test to identify loci with significant gene-by-environment interactions. Third, we compute the *m-value* of each study to identify in which studies a given variant has an effect and in which it does not. Forth, we visualize the result through a forest plot and PM-plot to understand the underlying nature of gene-by-environment interactions.

**Figure 1:**
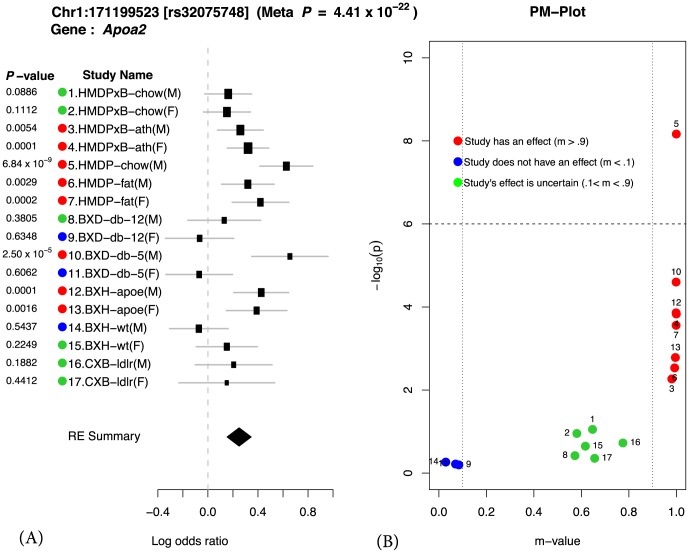
Application of Meta-GxE to *Apoa2* locus. The forest plot (A) shows heterogeneity in the effect sizes across different studies. The PM-plot (B) predicts that 7 studies have an effect at this locus, even though only 1 study (HMDP-chow(M)) is genome-wide significant with *P*-value.

We illustrate our methodology by examining a well-known region on mouse chromosome 1 harboring the *Apoa2* gene, which is known to be strongly associated with HDL cholesterol [23]. Figure 1 shows the results of applying our method to this locus. We first compute the effect size and its standard deviation for each of the 17 studies. These results are shown as a forest plot in Figure 1 (a). Second we compute the *P*-value for each individual study also shown in Figure 1 (a). If we were to follow traditional methodology and evaluate each study separately, we would declare an effect present in a study if the *P*-value exceeds a predefined genome-wide significance threshold (*P* 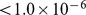). In this case, we would only identify the locus as associated in a single study, HMDP-chow(M) (*P* = 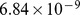). On the other hand, in our approach, we combine all studies to compute a single *P*-value for each locus taking into account heterogeneity between studies. This approach leads to increased power over the simple approach considering each study separately. The combined meta *P*-value for the *Apoa2* locus is very significant (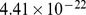), which is consistent with the fact that the largest individual study only has 749 animals compared to 4,965 in our combined study.

In order to evaluate how significantly different the effect sizes of the locus are between studies, we apply a heterogeneity test. The statistical test is based on Cochran's Q test [24], [25], which is a non-parametric test for testing if studies have the same effect or not. In this locus, the effect sizes are clearly different and not surprisingly the *P*-value of the heterogeneity test is significant (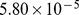). This provides strong statistical evidence of a gene-by-environment interaction at the locus. Below we more formally describe how heterogeneity in effect size at a given locus can be interpreted as gene-by-environment interaction.

If a variant is significant in the meta-analytic testing procedure, then this implies that the variant has an effect on the phenotype in one or more studies. Examining in which subset of the studies an effect is present and comparing to the environmental conditions of the studies can provide clues to the nature of gene-by-environment interactions at the locus. However, the presence of the effect may not be reflected in the study-specific *P*-value due to a lack of statistical power. Therefore, it is difficult to distinguish only by a *P*-value if an effect is absent in a particular study due to a gene-by-environment interaction at the locus or a lack of power. In order to identify which studies have effects, we utilize a statistic called the m-value [16], which estimates the posterior probability of an effect being present in a study given the observations from all other studies. We visualize the results through a PM-plot, in which *P*-values are simultaneously visualized with the m-values at each tested locus. These plots allow us to identify in which studies a given variant has an effect and in which it does not. M-values for a given variant have the following interpretation: a study with a small m-value(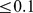) is predicted not to be affected by the variant, while a study with a large m-value(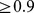) is predicted to be affected by the variant.

The PM-plot for the *Apoa2* locus is shown in Figure 1 (b). If we only look at the separate study *P*-values (y-axis), we can conclude that this locus only has an effect in HMDP-chow(M). However, if we look at m-value (x-axis), then we find 8 studies (HMDPxB-ath(M), HMDPxB-ath(F), HMDP-chow(M), HMDP-fat(M), HMDP-fat(F), BxD-db-5(M), BxH-apoe(M), BxH-apoe(F)), where we predict that the variation has an effect, while in 3 studies (BxD-db-12(F), BxD-db-5(F), BxH-wt(M)) we predict there is no effect. The predictions for the remaining 6 studies are ambiguous.

From Figure 1, we observe that differences in effect sizes among the studies are remarkably consistent when considering the environmental factors of each study as described in Table 1. For example, when comparing study 1–4, the effect size of the locus decreases in both the male and female HMDPxB studies in the chow diet (chow study) relative to the fat diet (ath study). Thus we can see that when the mice have Leiden/CETP transgene, which cause high total cholesterol level and high LDL cholesterol level, effect size of this locus on HDL cholesterol level in blood is affected by the fat level of diet. Similarly, when comparing study 12–15, the knockout of the *Apoe* gene affects the effect sizes for both male and female BxH crosses. However, in the BxD cross (study 8–11), where each animal is homozygous for a mutation causing a deficiency of the leptin receptor, the effect of the locus is very strong in the young male animals, while as animals get older and become fatter, the effect becomes weaker. However in the case of female mice, the effect of the locus is nearly absent at both 5 and 12 weeks of age. Thus we can see that sex plays an important role in affecting HDL when the leptin receptor activity is deficient. We note that there are many genes in this locus and the genetic mechanism of interactions may involve genes other than *Apoa2*. Despite this caveat, the results of Meta-GxE at this locus provides insights into the nature of GxE and can provide a starting point for further investigation.

**Table 1:** 17 HDL studies for meta analysis.

Study ID	Strains	Conditions	Age	Sex	# Strains	# Samples	# Sig Loci	Ref
HMDPxB-chow(M)	(HMDP×BL/6) F1	Leiden/CETP TG,chow diet	8 weeks	M	97	516	1	U
HMDPxB-chow(F)	(HMDP×BL/6) F1	Leiden/CETP TG, chow diet	8 weeks	F	95	468	0	U
HMDPxB-ath(M)	(HMDP×BL/6) F1	Leiden/CETP TG, highfat diet	24 weeks	M	97	408	0	U
HMDPxB-ath(F)	(HMDP×BL/6) F1	Leiden/CETP TG, highfat diet	24 weeks	F	93	457	3	U
HMDP-chow(M)	HMDP	chow diet	12 weeks	M	111	749	6	[18]
HMDP-fat(M)	HMDP	highfat diet	16 weeks	M	106	586	0	[14]
HMDP-fat(F)	HMDP	highfat diet	16 weeks	F	92	475	0	[44]
BxD-db-12(M)	(DBA×BL/6) F2	BXD db/db, chow diet	12 weeks	M	125	125	0	[45]
BxD-db-12(F)	(DBA×BL/6) F2	BXD db/db, chow diet	12 weeks	F	122	122	0	[45]
BxD-db-5(M)	(DBA×BL/6) F2	BXD db/db, chow diet	5 weeks	M	109	109	1	[45]
BxD-db-5(F)	(DBA×BL/6) F2	BXD db/db, chow diet	5 weeks	F	139	139	0	[45]
BxH-apoe(M)	(C3H×BL/6) F2	BXH Apoe -/-	24 weeks	M	161	161	0	[46]
BxH-apoe(F)	(C3H×BL/6) F2	BXH Apoe -/-	24 weeks	F	174	174	0	[46]
BxH-wt(M)	(C3H×BL/6) F2	BXH wildtype, highfat diet	20 weeks	M	164	164	0	[47]
BxH-wt(F)	(C3H×BL/6) F2	BXH wildtype, highfat diet	20 weeks	F	144	144	0	[47]
CxB-ldlr(M)	(BALB/cJ×BL/6) F2	CXB LDLR -/-, highfat diet	12 weeks	M	124	124	0	U
CxB-ldlr(F)	(BALB/cJ×BL/6) F2	CXB LDLR -/-, highfat diet	12 weeks	F	64	64	0	U

Seventeen HDL studies are combined in the meta analysis. U in the Ref column represents a data set that is not yet published. Mice for the HMDPxB panel were created by breeding females of the various HMDP inbred strains to males carrying transgenes for both Apoe Leiden and for human Cholesterol Ester Transfer Protein (CETP) on a C57BL/6 genetic background. The Leiden/CETP transgenes [48], [49] cause high total cholesterol level and high LDL cholesterol level in the circulation, along with reduced HDL cholesterol. BxD db/db denotes a population of F2 mice from a cross between C57BL/6 DBA/2 with homozygous deficiency in leptin receptor (db/db), which results in obese mice. BxH Apoe -/- denotes denotes a population of F2 mice from a cross between C57BL/6 and C3H also carrying a deficiency in apolipoprotein E. CxB LDLR -/- denotes a population of F2 mice from a cross between C57BL/6 and BALB/cBy also carrying a deficiency in LDL receptor, which results in high LDL cholesterol level in the circulation BXH wildtype denotes a population of F2 mice from a cross between C57BL/6 and C3H.

We note that an alternate explanation for differences in effect sizes between studies is the presence of gene-by-gene interactions and differences in the genetic backgrounds of the studies. While this is a possible explanation for differences in effect sizes between the different crosses and the HMDP studies, in Figure 1, we see many differences in effect sizes among studies with the same genetic background. Thus gene-by-gene interactions can only partially explain the differences in observed effect sizes.

### The connection between random effects meta-analysis and gene-by-environment interactions

Gene-by-environment interactions, random effects meta-analysis and heterogeneity testing are closely related. Suppose we have 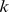 studies each conducted under different environmental conditions. We define the following linear model, where 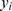 is the observed phenotype for study 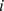, 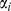 is the phenotype mean for study 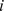, 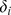 is the genetic effect on the phenotype for study 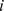, 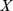 is the genotype, and 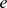 is the residual error.
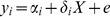
(1)


Since each environment is different, the effect size 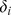 is partially determined by environmentally-specific factors and partially determined by factors common to all studies. Given that we can decompose the effect 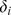 into environment-independent and environment-dependent factors. Then we define the following linear model, where 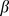 is the environment-independent genetic effect and 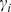 is the environment-dependent genetic effect for study 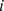.
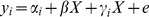
(2)


In order to test for the presence of an effect shared across environments, we test the null hypothesis 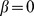 and to test for the presence of a gene-by-environment interaction, we test the hypothesis that 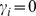.

In the random effects meta-analysis, we assume that the effect size 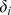 is sampled from a normal distribution with mean 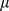 and variance 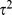, denoted 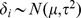. Under this assumption, we test the null hypothesis 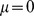 and 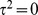, in order to obtain a study-wide *P*-value. Additionally, we perform a heterogeneity test to test the null hypothesis 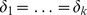 versus the alternative hypothesis 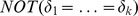. We posit that by conducting hypotheses tests in the meta-analysis framework, we are simultaneously testing for the presence of environmentally-independent and environmentally-specific effects and that by applying heterogeneity testing we are testing for only environmentally-specific effects.

Consider that in the meta-analysis framework 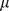 is analogous to 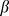 and the variation (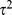) around 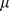 is analogous to variation among 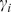
s. In the random effects meta analysis testing framework we are testing if 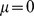 and 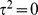. This is equivalent to testing both environmentally-independent (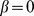) and environmentally-dependent (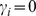) effects simultaneously. In heterogeneity testing, we test the null hypothesis 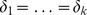 versus the alternative hypothesis 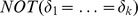. When the environmentally-dependent effect (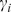) is 0 it means that 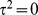 and thus 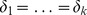. When 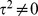, we expect that 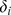 will vary around 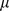, so that we do not expect that 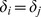. Since the variation (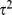) of 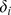 around 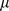 is analogous to the variable 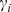, heterogeneity testing in the meta-analysis framework is approximately equivalent to testing for environmentally-specific effects.

### Gene-by-environment interactions are prevalent in mouse association studies

The presence of heterogeneity in the effect size at causal genetic loci due to gene-by-environment interactions is naturally expected in mouse genetic studies when combining studies with varying environmental conditions. One extreme example comes from a knock-out experiment. If the knocked-out gene is causal for a particular trait, then we can expect that the gene would have no effect on a knock-out mouse, while the gene would have an effect on the wild type mouse. This is a binary form of heterogeneity. In a less extreme form of heterogeneity, the effect of a given gene may be affected by an environmental factor which varies in different mice – ranging from small effects to large effects.

To see the relationship between significance of the association and gene-by-environment interactions, we compute and compare this *P*-value for each SNP from the 17 studies using the random effects meta-analysis to a measure of heterogeneity. Heterogeneity can be assessed by 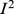 statistic, which describes the percentage of variation across studies that is due to heterogeneity rather than chance [26].

**Figure 2:**
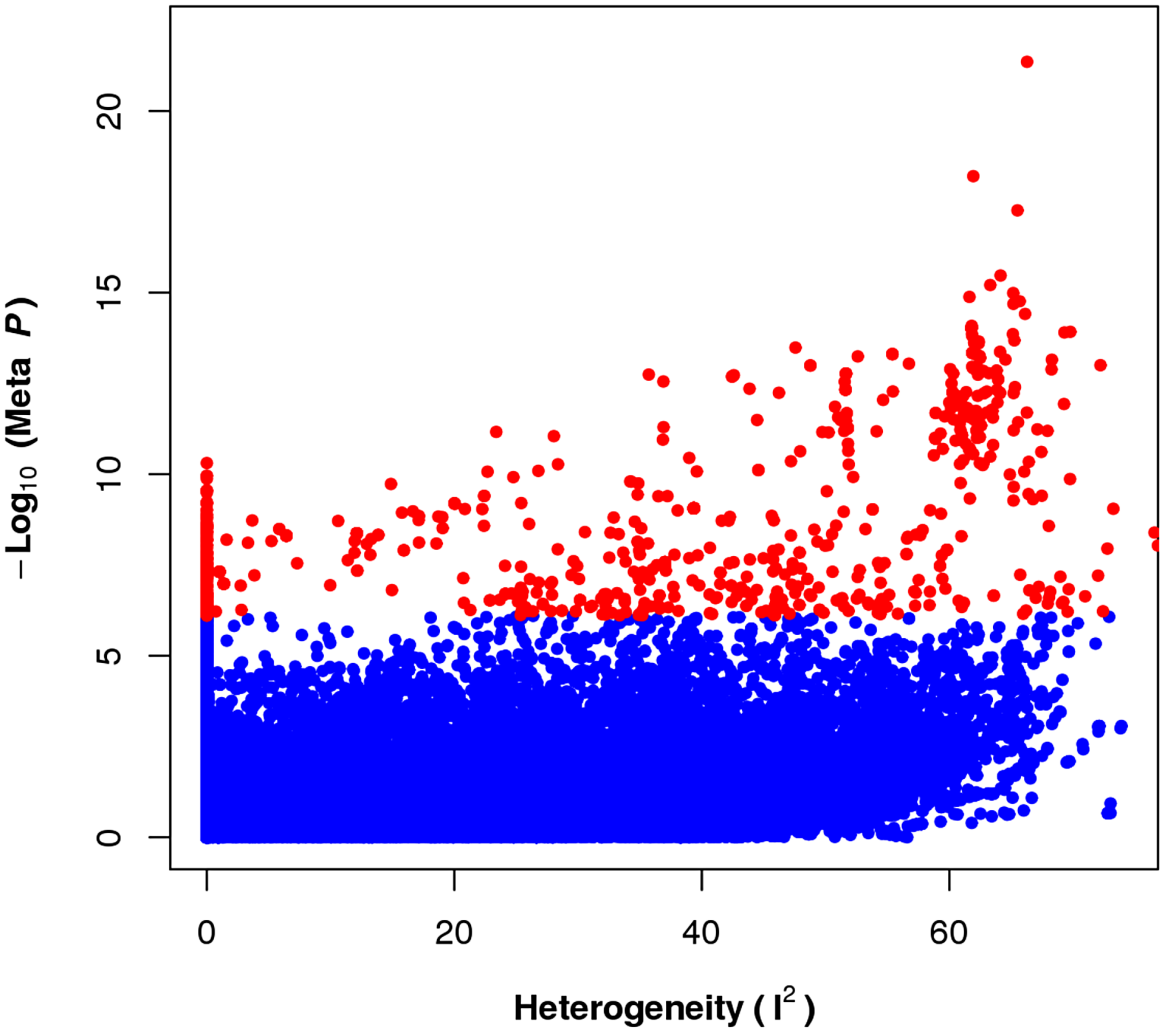
The prevalence of heterogeneity in effect size of significant loci. Each dot represents association between SNPs and HDL phenotype from applying random effects based meta-analysis approach. Dots with larger 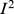 value represents the existence of more heterogeneity at the locus between studies. The distribution of the heterogeneity statistic for significant SNPs (red dots) in the meta analysis is skewed toward higher heterogeneity while the non-significant SNPs are much less skewed.

Figure 2 compares 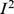 statistic with the meta-analysis *P*-value for each SNP. In this figure, we see that 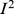 is uniformly distributed for the non-significant SNPs (blue dots), while it is right skewed for significant SNPs (red dots), indicating that more significant SNPs have a greater potential for exhibiting heterogeneity in effect. Since heterogeneity in this case can be interpreted as representing gene-by-environment interactions, as heterogeneity is induced by differences in the environment, we see that the presence of a GxE interaction confers higher power to detect an association.

### Power of meta-analysis for detecting gene-by-environment interactions

**Figure 3:**
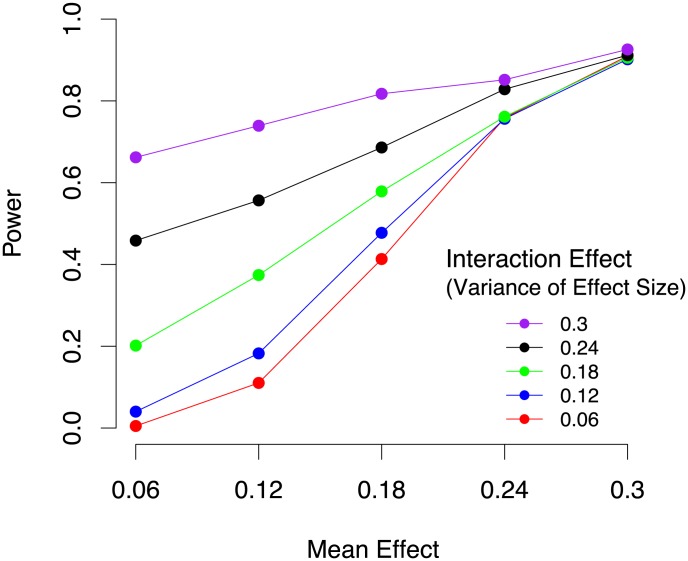
Power of mouse meta-analysis to identify gene-by-environment interactions in 4,965 animals from 17 studies under varying mean effect sizes and the per study variance of the effect size which corresponds to gene-by-environment effects.

The power to identify both gene-by-environment and main effects in a meta-analysis of mouse studies depends on both the main effect size and the amount of heterogeneity. We performed simulations using the genotypes of the 17 mouse studies analyzed in this paper. We simulated a range of main effect (mean effect) sizes and a range of gene-by-environment effects. We are simulating the realistic scenario in which we do not know exactly the set of covariates which are responsible for the gene-by-environment effects. We simulated gene-by-environment effects by drawing the effect in each study from a distribution with a mean given by the main effect size and a variance controlling the magnitude of gene-by-environment interactions. If this variance is small, then all of the studies have close to the same effect size and there are few gene-by-environment effects. If the variance is high, then there are strong gene-by-environment effects. Figure 3 shows the results of our simulations. 1000 simulated phenotypes were generated for each mean and variance pair. Statistical power is estimated by computing the proportion of the datasets in which a simulated effect is detected. We observe that the power is high for a wide range of main effect sizes and gene-by-environment effect sizes which is explained by the large sample size of the study. We also observe that even for small main effects, if there are strong gene-by-environment effects, we can still identify the locus. This is because in this case a subset of the studies will have strong effect sizes due to gene-by-environment effects.

Our approach is not the only way to analyze a meta-analysis study. We compare the power to two other meta-analytic approaches. The first is the traditional meta-analysis strategy which uses a fixed effects model (FE) in which all of the effect sizes across studies are assumed to be the same. We utilize an extension of the fixed effects model which corrects for population structure [15]. A second alternate strategy is to simply apply the heterogeneity test (HE), which in our framework is only applied to loci first identified using random effects meta-analysis. The HE test follows the intuition that loci with high heterogeneity will harbor gene-by-environment interactions. For the purposes of the comparison we refer to Meta-GxE as the random effects (RE) model.

**Figure 4:**
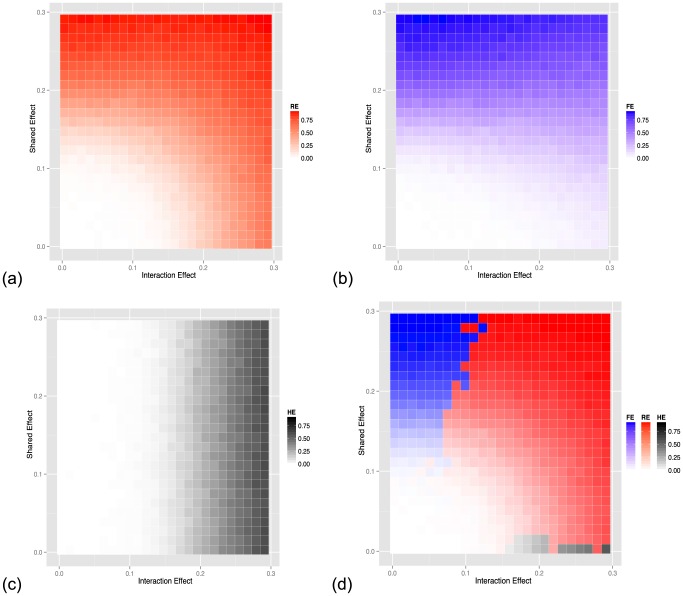
Power of (a) random-effect, (b) fixed-effect meta-analysis and (c) heterogeneity meta-analysis methods as a function of the effect size and the strength of the interaction effect (heterogeneity). (d) shows a comparison of the three methods with the color corresponding to the method with the highest power.

The level of gene-by-environment interactions can be simulated by changing both the environment-dependent and environment-independent effect simultaneously, when simulating the phenotype. Figure 4 (a)–(c) shows the power of the three approaches (RE, FE, HE) respectively when we vary the mean and variance of the effect size distribution we sampled from. In this simulation study, mean effect represents shared effect and variance of the effect size represents interaction effect. As expected, RE has high power in cases where the shared effect or the interaction effect is large. FE has high power when the shared effect is large and the HE test has high power when the interaction effect is large. Figure 4 (d) shows the heatmap which is colored with the color of highest powered approach. FE is most powerful at the top-left region, HE is most powerful at the bottom-right region, while RE is most powerful for a majority of the simulations. In the Text S1, we show through simulations that our methodology outperforms the alternative fixed effects and heterogeneity testing approaches when the effect is present in a subset of the studies, which is another possible interaction model we can assume. We also show in the Text S1 that our approach is more powerful than the traditional uni- or multi-variate gene-by-environment association approach which assumes knowledge of the covariates involved in gene-by-environment interactions. For the traditional uni- or multi-variate approach, required knowledge includes kinds of variable (e.g. sex, age, gene knockouts) and encoding of the variables (e.g. binary values, continuous values). In the Text S1, we also show the our proposed approach controls the false positive rate.?

### Application to 17 mouse HDL studies

We applied Meta-GxE to 17 mouse genetic studies conducted under various environmental conditions where each study measured HDL cholesterol. Table 1 summarizes each study. More details are provided in the Materials and Methods section and in Text S1. We analyzed all 17 studies together and we also analyzed the 9 male and 8 female studies separately. Some significant associations are shared and some associations are specific to males and females.

**Figure 5:**
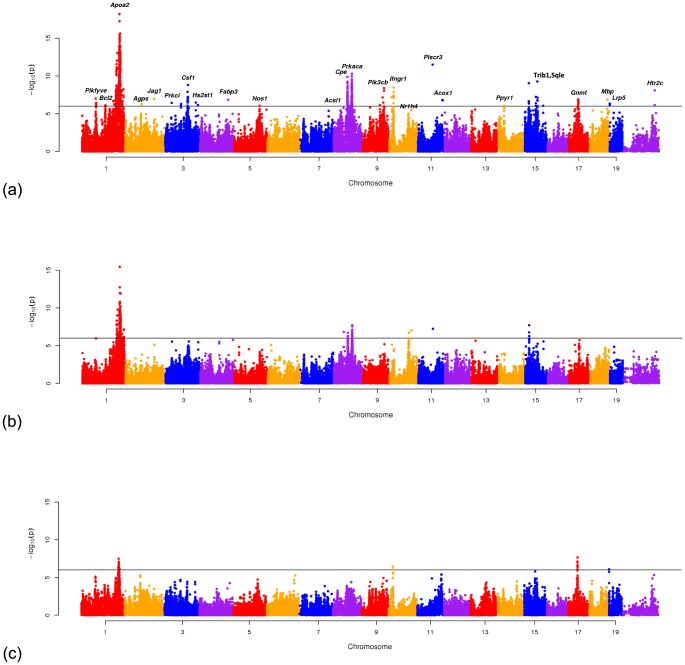
Manhattan plots showing the results of Meta-GxE applied to (a) 17 HDL studies, (b) 9 HDL studies consisting only of male animals and (c) 8 studies consisting only of female animals.

The Manhattan plots in Figure 5 show the meta-GxE result when applied to the 17 studies, 9 male only studies and 8 female only studies. Table 2 summarizes 26 significant peaks (P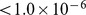) showing the *P*-values obtained by applying meta-GxE to the male only studies (9 studies), the female only studies (8 studies) and the male+female studies (17 studies). For each significant locus, we computed m-values, interpreted as the posterior probability of having an effect on the phenotype and report the number of studies with an effect (E), the number of studies with ambiguous effect size (A) and the number of studies without an effect (N). We also report the number of individual studies where the locus was significant (*P* 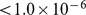). As seen in the table, many of the loci were not significant in any of the individual studies and would not have been discovered without combining the studies. We note that we use a more stringent genome wide threshold of *P* 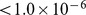 than was used in the original studies. The Genes in Region and Gene Refs columns contain the gene names near the locus previously known to affect HDL cholesterol level or closely related lipid level in the blood and associated literature citations.

**Table 2:** 26 significant loci identified by applying Meta-GxE analysis.

SNP	Meta GxE *P*	Meta GxE *P*	Meta GxE *P*	# of Studies w/	HE Meta *P*	# Studies	Genes	Gene
Location	(Male)	(Female)	(Male+Female)	Significant *P*	(Male+Female)	E/A/N	in Region	Refs
Chr1:64752822 (rs31078051)	1.12×10^−6^	2.37×10^−3^	**9.82×10^−8^**	0	3.82×10^−3^	2/14/1	**Pikfyve**	[50]
Chr1:107271282 (rs32203839)	6.69×10^−4^	2.66×10^−4^	**7.67×10^−7^**	0	7.55×10^−2^	6/0/11	**Bcl2**	[51]
Chr1:171199523 (rs32075748)	**3.45×10^−16^**	**1.39×10^−7^**	**4.41×10^−22^**	1	5.80×10^−5^	8/6/3	**Apoa2**	[52]
Chr2:77837584 (rs6273567)	1.55×10^−4^	7.17×10^−4^	**5.74×10^−7^**	0	2.25×10^−2^	3/14/0	**Agps**	[53]
Chr2:134421733 (rs27238693)	7.69×10^−6^	1.66×10^−3^	**1.09×10^−7^**	0	1.63×10^−2^	5/12/0	**Jag1**	[54]
Chr3:32944259 (rs29869794)	2.97×10^−6^	2.49×10^−2^	**3.68×10^−7^**	0	2.36×10^−5^	3/8/6	**Prkci**	[55]
Chr3:76066632 (rs31487078)	5.89×10^−3^	2.29×10^−5^	**5.03×10^−7^**	0	6.56×10^−2^	4/13/0	**Novel**	-
Chr3:107430396 (rs30013147)	9.59×10^−6^	3.84×10^−5^	**1.56×10^−9^**	0	7.74×10^−2^	7/10/0	**Csf1**	[56]
Chr3:143466942 (rs30206761)	1.82×10^−3^	3.97×10^−5^	**3.34×10^−7^**	0	8.60×10^−2^	7/10/0	**Hs2st1**	[57]
Chr4:131925523 (rs32595861)	1.72×10^−4^	2.84×10^−4^	**1.42×10^−7^**	0	8.65×10^−4^	6/8/3	**Fabp3**	[58]
Chr5:119034507 (rs33131194)	1.23×10^−4^	2.59×10^−3^	**9.00×10^−7^**	0	3.94×10^−1^	9/8/0	**Nos1**	[59], [60]
Chr8:46903188 (rs33272858)	**1.47×10^−7^**	6.52×10^−1^	1.66×10^−6^	1	1.62×10^−4^	2/11/4	**Acsl1**	[61]
Chr8:64150094 (rs31750594)	**1.96×10^−7^**	1.89×10^−4^	**1.33×10^−10^**	0	8.34×10^−1^	11/6/0	**Cpe**	[62]
Chr8:84073148 (rs33435859)	**1.95×10^−8^**	4.53×10^−4^	**4.94×10^−11^**	0	8.33×10^−1^	12/5/0	**Prkaca**	[27]
Chr9:101972687 (rs6333310)	1.22×10^−4^	1.22×10^−5^	**4.05×10^−9^**	0	**1.98×10^−8^**	2/1/14	**Pik3cb**	[63]
Chr10:21399819 (rs29363941)	9.07×10^−4^	**3.64×10^−7^**	**3.36×10^−9^**	0	1.18×10^−2^	3/12/2	**Ifngr1**	[64]
Chr10:90146088 (rs29370592)	**1.93×10^−7^**	0.756	1.02×10^−5^	1	8.94×10^−4^	2/14/1	**Nr1h4**	[65]
Chr11:69906552 (rs29477071)	**5.77×10^−8^**	1.35×10^−5^	**3.17×10^−12^**	0	**2.37×10^−9^**	6/9/2	**Plscr3**	[66]
Chr11:114083173 (rs29416888)	1.10×10^−4^	7.83×10^−5^	**1.71×10^−7^**	0	5.28×10^−5^	3/13/1	**Acox1**	[67]
Chr14:33632464 (rs31061259)	1.96×10^−4^	1.65×10^−3^	**8.90×10^−7^**	0	2.02×10^−5^	3/10/4	**Ppyr1**	[68]
Chr15:21194226 (rs31670969)	**1.96×10^−8^**	1.29×10^−2^	**8.97×10^−10^**	1	**5.65×10^−7^**	3/2/12	**Novel**	-
Chr15:59860191 (rs3718217)	5.64×10^−6^	1.45×10^−5^	**5.31×10^−10^**	0	9.92×10^−5^	5/10/2	**Trib1, Sqle**	[69], [70]
Chr17:46530712 (rs33259313)	1.09×10^−5^	4.90×10^−3^	**3.26×10^−7^**	0	3.53×10^−5^	5/10/2	**Gnmt**	[71]
Chr18:82240606 (rs13483466)	2.05×10^−4^	2.23×10^−4^	**1.32×10^−7^**	0	9.05×10^−1^	5/12/0	**Mbp**	[72]
Chr19:3319089 (rs31004232)	5.58×10^−2^	**8.53×10^−7^**	**4.56×10^−7^**	0	1.08×10^−1^	3/14/0	**Lrp5**	[73]
ChrX:151384614 (rs31202008)	2.59×10^−4^	4.72×10^−6^	**8.09×10^−9^**	0	1.12×10^−1^	5/5/0	**Htr2c**	[74]

Twentysix significant loci identified by applying Meta-GxE analysis of both random effects meta-analysis and heterogeneity testing to 17 mouse HDL studies under different environments containing 4,965 total animals. # studies E denotes the number of studies with an effect on HDL phenotype. # studies N denotes the number of studies with no effect on HDL phenotype. # studies A denotes the number of studies with an ambiguous effect size. Genes in region denotes candidate genes for each locus based on close proximity to the peak SNP and previously suggested role in lipid or apolipoprotein metabolism: **Pikfyve** (phosphoinositide kinase), **Bcl2** (B cell leukemia/lymphoma 2), **Apoa2** (apolipoprotein A-II), **Agps** (alkylglycerone phosphate synthase), **Jag1** (jagged 1), **Prkci** (protein kinase C), **Prkci** (colony stimulating factor 1 (macrophage)), **Hs2st1** (heparan sulfate 2-O-sulfotransferase 1), **Fabp3** (fatty acid binding protein 3), **Nos1** (nitric oxide synthase 1), **Acsl1** (acyl-CoA synthetase long-chain family member 1), **Cpe** (carboxypeptidase E), **Prkaca** (protein kinase, cAMP dependent, catalytic, alpha), **Acox1** (peroxisomal acyl-coenzyme A oxidase 1), **Ppyr1** (pancreatic polypeptide receptor 1), **Trib1**(tribbles homolog 1), **Sqle** (squalene epoxidase), **Gnmt** (glycine N-methyltransferase), **Mbp**(myelin basic protein), **Lrp5** (low density lipoprotein receptor-related protein 5), **Htr2c** (5-hydroxytryptamine (serotonin) receptor 2C).

Among the 26 loci that we identified by applying Meta-GxE, 24 loci are near the genes (mostly genes are located within 1MB of the peak) known to affect HDL or closely related lipid level in the blood, while 3 loci are novel.

For example, we identified 3 loci (Chr10:21399819, Chr19:3319089, ChrX:151384614) female mice show a more significant effect on HDL phenotypes than male mice. We also identified 7 loci (Chr1:171199523, Chr8:46903188, Chr8:64150094, Chr8:84073148, Chr10:90146088, Chr11:69906552, Chr15:21194226) where male mice show a more significant effect on HDL than female mice.

Interestingly, we observed that in 3 loci (Chr10:21399819, Chr19:3319089, ChrX:151384614), female mice are more highly affected, while in 7 loci (Chr1:171199523, Chr8:46903188, Chr8:64150094, Chr8:84073148, Chr10:90146088, Chr11:69906552, Chr15:21194226) male mice are more highly affected. Among 26 loci, many show a significant heterogeneity in effect sizes between the 17 studies, which we interpret as gene-by-environment interactions.

**Figure 6:**
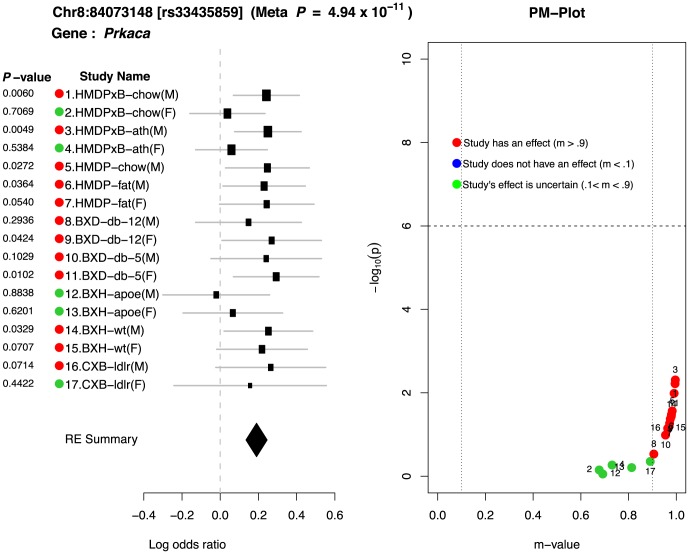
Peak SNP in chromosome 8 shows interesting gene-by-environment interactions between sex×mutation-driven LDL levels.

One interesting example showing strong gene-by-environment interaction is a locus in Chr8:84073148. This locus is located near the gene 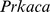, which is known to affect the abnormal lipid levels in blood [27]. Figure 6 shows the forest plot and PM-plot for this locus. If we look at the forest plot of the locus in Figure 6, we can easily see that there are two groups: 12 studies with an effect (red dots) and 5 studies with an ambiguous prediction of the existence of an effect (green dots). Interestingly, the log odds ratios of effect size for the 12 studies with an effect is about the same (around 0.2). The common characteristic in 4 of the 5 studies (HMDPxB-chow(F), HMDPxB-ath(F), BXH-apoe(F), CXB-ldlr(F)) is that they are female mice with high LDL levels in the blood. In addition, in all 4 cases, these high LDL levels are caused by mutant genes. Mice in HMDPxB-chow and HMDPxB-ath studies have transgenes for both Apoe Leiden and for human Cholesterol Ester Transfer Protein (CETP), while mice in the BXH-apoe and CXB-ldlr studies carried knockouts of the genes for Apoe and LDL receptor, respectively. This is a strong evidence that there is an interaction between sex×mutation-driven LDL levels through this locus (Chr8:84073148) when affecting HDL levels in mice.

Figures S5, S6, S7, S8, S9, S10, S11, S12, S13, S14, S15, S16, S17, S18, S19, S20, S21, S22, S23, S24, S25, S26, S27, S28, S29, S30 show the forest plots and PM-plots for each locus, which show information such as effect sizes, the direction of the effect, which study has an effect and which study does not have an effect for each of 17 studies at the given locus.

## Discussion

In this paper, we present a new meta-analysis approach for discovering gene-by-environment interactions that can be applied to a large number of heterogeneous studies each conducted in different environments and with animals from different genetic backgrounds. We show the practical utility of the proposed method by applying it to 17 mouse HDL studies containing 4,965 mice, and we successfully identify many known loci involved in HDL. Consistent with the results of meta-analysis in human studies, our combined study finds many loci which were not discovered in any of the individual studies.

A point of emphasis is that in our study design, in each of the combined studies, all of the individuals in the study are subject to only a single environment. This is distinct from other approaches for discovery of gene-by-environment interactions using meta-analysis such as those described in [28]. In these approaches, in each of the combined studies, the individuals in the study are subject to multiple environments and information on each individual's environment is collected. Gene-by-environment statistics are then computed in each study and then combined in the meta-analysis. In our study design, we compute main effect sizes for each SNP and then look for variants where the effect sizes are different suggesting the presence of a gene-by-environment interaction.

In our meta-analysis approach, we assume that we do not have any prior knowledge of the effect size in any particular study. However one might incorporate prior knowledge of the specific environmental effects. In some cases, one might know that some of the studies have similar effect sizes as compared to others. Or the prior knowledge might suggest that one specific study needs to be eliminated in the meta analysis. If we utilize such prior knowledge, we may be able to achieve even higher statistical power.

In this paper we have addressed how to perform meta-analysis when the studies have different genetic structures, building off the results of our previous study [15]. While in this paper we combine 7 HMDP studies with 10 genetic crosses, the approach in principle can be used to combine any variety of study types. Recently, several strategies for mouse genome-wide association mapping have been proposed [29] [17]. These include HMDP [18], collaborative cross [30] and outbredstock [21] [17]. The approach presented here can be utilized to combine these different kinds of studies and is a roadmap for integrating the results of different strategies for mouse GWAS.

Although we have focused on explaining heterogeneity by gene-by-environment interaction, it is possible that the differences in effect sizes can be caused by gene-by-gene interactions on different genetic backgrounds, where the interacting variants differ in frequency in the different studies. While gene-by-gene interactions certainly contribute to locus heterogeneity, we predict that, in combining studies with similar genetic structures, locus heterogeneity more likely arises from gene-by-environment interactions. In any case, determining whether or not these heterogeneous loci are environment-driven or interaction-driven is an important and interesting direction for future study.

## Materials and Methods

### Standard study design for testing gene-by-environment interactions

In the model organism studies for which we can control the environment, the standard study design for testing gene-by-environment interactions is to combine multiple cohorts whose environments are known. The environmental value that we vary is typically a quantitative measure that we can model with a single random variable. Thus, the standard univariate linear model can be applied
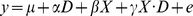
where 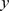 is 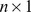 vector of phenotype measurements from 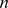 individuals, 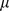 is the phenotype mean, 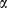 is the main environmental effect mean, 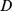 is 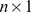 environmental status vector, 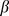 is the genetic effect, 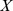 is 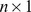 genotype vector, 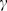 is GxE interactions effect,  denotes the dot-product between two vectors, and 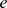 is the residual error, which follows normal distribution. In this model, vector 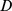 is a vector of indicators which describes the environmental status of each individual. study. For example, Suppose the environmental condition of one study is wildtype and that of another is gene knockout. In this case, the environmental condition of wildtype is described as 0 and that of knockout is described as 1. In order to test if there are interactions, we test the null hypothesis 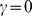 versus the alternative hypothesis 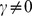. Another possible testing strategy is to test the interactions effect together with the genetic effect, that is, the null hypothesis 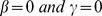 versus the alternative hypothesis 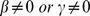. This strategy is powerful in detecting loci exhibiting both the genetic effects and the interactions effects.

### Multivariate interactions model

For more complicated scenarios where the different environments can not be modeled with a single variable, a straightforward extension of the standard univariate interactions model is the multivariate model. Suppose that there are k different possible environments and the information on the environments of each individual are captured by a matrix D which has k columns where each column corresponds to one environment. Then, the standard multivariate interactions model will be
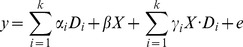
(3)

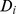
 is the 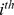 column of the D matrix, 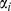 is the environment specific mean, 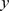 denotes the phenotype measurements, 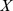 denotes the genotypes, 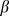 denotes the fixed genetic effect, 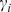 denotes GxE interactions effect of 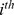 environmental variable and, and 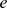 denotes the residual error. Then the testing will be between the null hypothesis 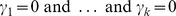 versus the alternative hypothesis 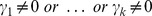. The test statistic will be
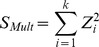
where 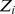 is the z-score corresponding to 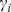. 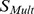 follow 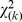 under the null. Similarly to the univariate model, if we want to test the interactions effect together with genetic effect, we add the z-score corresponding to 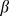 into the statistic, in which case the statistic will follow 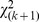.

### Standard meta-analysis approach

Before we describe the relationship between gene-by-environment interactions and meta-analysis, we first describe the standard fixed effects and random effects meta-analysis in details.

#### Fixed effects model meta analysis

In standard meta-analysis, we have 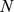 studies. In each of the 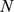 studies, we estimate the effect size of interest. Suppose that we estimate the genetic effect in study 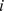,
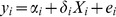
(4)


We can obtain the estimates of 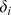 and its variance 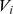. In the fixed effects model meta-analysis, we assume that the underlying effect sizes are the same as 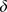 (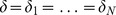). The best estimate of 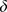 is the inverse-variance weighted effect size,
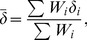
(5)where 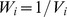 is the so-called inverse variance. Then we test the null hypothesis 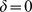 versus the alternative hypothesis 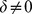.

#### Testing heterogeneity

The phenomenon that the underlying effect sizes differ between studies is called *heterogeneity*. The presence of heterogeneity is tested using the Cochran's Q test [24], [25]. Cochran's Q test is a non-parametric test for testing if N studies have the same effect or not. Particularly it tests the null hypothesis 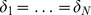 versus the alternative hypothesis 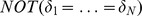. Cochran's Q statistic can be calculated as the weighted sum of squared differences between individual study effects and the pooled effect across studies.
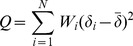
(6)Cochran's Q statistic has a chi-square statistic with 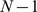 degrees of freedom.

#### Random effects model meta analysis

Under the random effects model meta-analysis, we explicitly model heterogeneity by assuming a hierarchical model. We assume that the effect size of each study 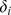 is a random variable sampled from a distribution with amean 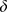 and variance 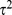,
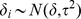
Traditional formulations of a random effects meta-analysis method are known to be overly conservative [24], [31], [32]. However, we recently developed a random effects model that addresses this issue [33]. The method assumes that there is no heterogeneity under the null, a modification that is natural in the context of association studies because the effect size should be fixed to be zero under the null hypothesis. This random effects model tests the null hypothesis 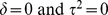 versus the alternative hypothesis 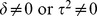.

Similarly to the traditional random effects model [24], we use the likelihood ratio framework considering each statistic as a single observation. Since we assume no heterogeneity under the null, 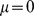 and 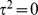 under the null hypothesis. The likelihoods are then
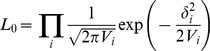


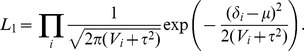
The maximum likelihood estimates 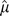 and 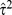 can be found by an iterative procedure suggested by Hardy and Thompson [34]. Then the likelihood ratio test statistic can be built
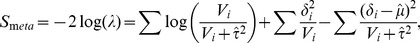
(7)whose *P*-value is calculated using tabulated values [33].

### Relation between gene-by-environment interactions and meta-analysis

Here we explain more about the relationship between gene-by-environment interactions and meta-analysis based on the explanation in Results section. If we do not consider the interactions, it has been already known that the fixed effects model meta-analysis is approximately equivalent to the linear model of combined cohorts [35]. That is, the fixed effects model equation (5) gives approximately equivalent results to the combined linear model
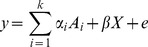
(8)where 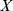 is the combined genotype vector from all cohorts, 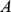 is a matrix that includes indicator columns which identify which individual is in each cohort, 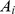 is the 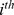 column of matrix A, and 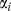 is the cohort specific mean. The two methods are approximately equivalent because they both test the fixed mean effect (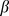 in equation (8) and 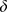 in equation (5)). The subtle difference between the two models is that in equation (8), we assume the error 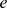 follows a single normal distribution (e.g. 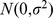), whereas in equation (5), the variance of the distributions may differ between studies (e.g. 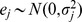 for each 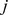). In other words, under the constant error variance assumption (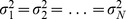), the two models become equivalent and 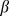 in equation (8) equals 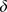 in equation (5),
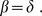



Similarly, by considering interactions, we extend this argument to show the relationship between gene-by-environment interactions and meta-analysis. We consider the relationship between equation (3) and equation (4). For simplicity of the notation, we consider the case where the matrix D is defined in such a way that each individual is only in one environment such that the D matrix is equivalent to the matrix A described above. If we assume the constant error variance assumption, we establish the following relationship,
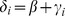
where the left hand side is the coefficient of the genotype 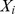 of study 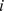 from the meta-analysis equation (4) and the right hand side is the same coefficient of 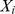 (the study 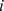
's part within the combined genotype matrix 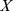) from the equation (3).

Suppose that there are no interactions (null hypothesis of interaction testing). Then, 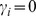 for each study 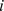. Thus, the effect size of meta-analysis 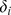 is equivalent to 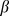, the genetic effects that are invariant across studies. Therefore, 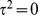 (null hypothesis of heterogeneity testing). On the other hand, suppose that 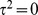 (null hypothesis of heterogeneity testing). Naturally, 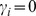 for all studies (null hypothesis of interaction testing). This shows that the null hypothesis of the interactions test in the model (3) and the null hypothesis of the heterogeneity test in meta-analysis are equivalent. As a result, we can utilize meta-analytic heterogeneity testing to detect interactions.

Using reasoning, it is straightforward to show that we can utilize the random effects model meta-analysis method to detect the mean effect and the interaction effect at the same time, which can be powerful for identifying loci bearing both kinds of effects.

### Controlling for population structure within studies

Model organism such as the mouse are well-known to exhibit population structure or cryptic relatedness [36], [37], where genetic similarities between individuals both inhibit the ability to find true associations and cause the appearance of a large number of false or spurious associations. Mixed effects models are often used in order to correct this problem [38]–[42]. Methods employing a mixed effects correction account for the genetic similarity between individuals with the introduction of a random variable into the traditional linear model.
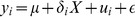
(9)


In the model in equation (9), the random variable 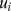 represents the vector of genetic contributions to the phenotype for individuals in population 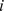. This random variable is assumed to follow a normal distribution with 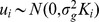, where 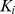 is the 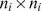 kinship coefficient matrix for population 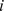. With this assumption, the total variance of 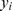 is given by 
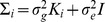
. A z-score statistic is derived for the test 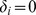 by noting the distribution of the estimate of 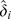. In order to avoid complicated notation, we introduce a more basic matrix form of the model in equation (9), shown in equation (10).
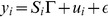
(10)


In equation (10), 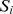 is a 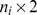 matrix with the first column being a vector of 1 s representing the global mean and the second vector is the vector and 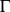 is a 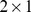 coefficient vector containing the mean 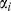 and genotype effect 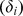. We note that this form also easily extends to models with multiple covariates. The maximum likelihood estimate for 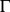 in population 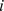 is given by 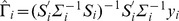 which follows a normal distribution with a mean equal to the true 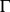 and variance 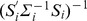. The estimates of the effect size 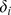 and standard error of the 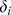 (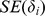) are then given in equation (11) and equation (12), where 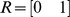 is a vector used to select the appropriate entry in the vector 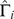.
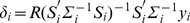
(11)

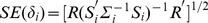
(12)


### Meta-analysis of studies with population structure

When we test gene-by-environment interactions with meta analysis approaches, one important step is correcting for population structure. This can be achieved by correcting for population structure within each study first as described above. For example, consider the random effects model meta-analysis method that we primarily focus on. We employ population structure control, using (11) and (12). Then the likelihood ratio test statistic will be
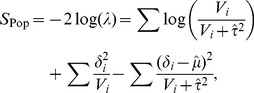
(13)where 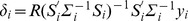 and 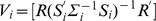.

### Identifying studies with an effect

After identifying loci exhibiting interaction effects, we employ the meta-analysis interpretation framework that we recently developed. The *m-value* [16] is the posterior probability that the effect exists in each study. Suppose we have 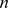 number of studies we want to combine. Let 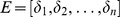 be the vector of estimated effect sizes and 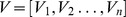 be the vector of estimated variance of 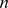 effect sizes. We assume that the effect size 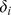 follows the normal distribution.
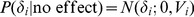
(14)

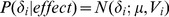
(15)We assume that the prior for the effect size is
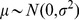
(16)A possible choice for 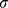 in GWASs is 0.2 for small effect and 0.4 for large effect [43]. We also denote 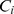 be a random variable whose value is 1 if a study 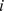 has an effect and 0 otherwise. We also denote 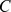 as a vector of 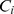 for 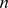 studies. Since 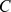 has 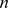 binary values, 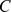 can be 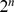 possible configurations. Let 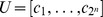 be a vector containing all the possible these configurations. We define *m-value* 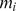 as the probability 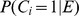, which is the probability of study 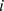 having an effect given the estimated effect sizes. We can compute this probability using the Bayes' theorem in the following way.
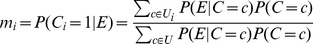
(17)where 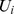 is a subset of 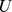 whose elements' 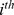 value is 1. Now we need to compute 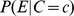 and 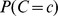. 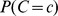 can be computed as
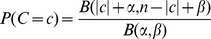
(18)where 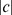 denotes the number of 1's in c and B denotes the beta function and we set 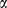 and 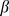 as 1 [16]. The probability 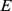 given configuration 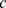, 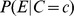, can be computed as
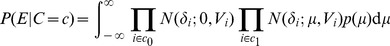
(19)

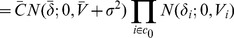
(20)

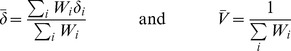
(21)where where 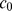 is the indices of 0 in 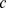 and 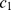 is the indices of 1 in 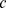, 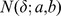 denotes the probability density function of the normal distribution with mean 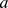 and variance 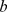. 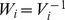 is the inverse variance or precision and 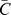 is a scaling factor.
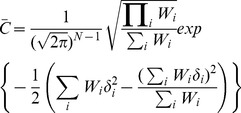
(22)All summations appeared for computing 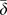, 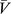 and 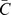 are with respect to 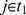.

The *m-value*s have the following interpretations: small *m-value*s(0.1) represent a study that is predicted to not have an effect, large *m-value*s(0.9) represent a study that is predicted to have an effect, otherwise it is ambiguous to make a prediction. It was previously reported that *m-value*s can accurately distinguish studies having an effect from the studies not having an effect [16]. For interpreting and understanding the result of the meta-analysis, it is informative to look at the *P*-value and m-value at the same time. We propose to apply the PM-plot framework [16], which plots the *P*-values and m-values of each study together in two dimensions. Figure 1 (b) shows one example of a PM-plot. In this example, studies with an *m-value* less than 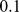 are interpreted as studies not having an effect while studies with an *m-value* greater than 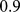 are interpreted as studies having an effect. For studies with an *m-value* between 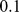 and 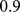, we cannot make a decision. One reason that studies are ambiguous (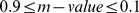) is that they are underpowered due to small sample size. If the sample size increases, the study can be drawn to either the left or the right side.

## Supporting Information

Figure S1 Power comparison between random-effect, fixed-effect meta-analysis and heterogeneity testing.
(EPS)

Figure S2 The association result of the fixed effects meta analysis.
(EPS)

Figure S3 Power comparison between random-effect meta-analysis and traditional wald test based approach.
(EPS)

Figure S4 Power comparison between heterogeneity testing approach and traditional wald test based approach.
(EPS)

Figure S5 Forest plot and PM-plot for Chr1:64752822 locus.
(EPS)

Figure S6 Forest plot and PM-plot for Chr1:107271282 locus.
(EPS)

Figure S7 Forest plot and PM-plot for Chr1:171199523 locus.
(EPS)

Figure S8 Forest plot and PM-plot for Chr2:77837584 locus.
(EPS)

Figure S9 Forest plot and PM-plot for Chr2:134421733 locus.
(EPS)

Figure S10 Forest plot and PM-plot for Chr3:32944259 locus.
(EPS)

Figure S11 Forest plot and PM-plot for Chr3:76066632 locus.
(EPS)

Figure S12 Forest plot and PM-plot for Chr3:107430396 locus.
(EPS)

Figure S13 Forest plot and PM-plot for Chr3:143466942 locus.
(EPS)

Figure S14 Forest plot and PM-plot for Chr4:131925523 locus.
(EPS)

Figure S15 Forest plot and PM-plot for Chr5:119034507 locus.
(EPS)

Figure S16 Forest plot and PM-plot for Chr8:46903188 locus.
(EPS)

Figure S17 Forest plot and PM-plot for Chr8:64150094 locus.
(EPS)

Figure S18 Forest plot and PM-plot for Chr8:84073148 locus.
(EPS)

Figure S19 Forest plot and PM-plot for Chr9:101972687 locus.
(EPS)

Figure S20 Forest plot and PM-plot for Chr10:21399819 locus.
(EPS)

Figure S21 Forest plot and PM-plot for Chr10:90146088 locus.
(EPS)

Figure S22 Forest plot and PM-plot for Chr11:69906552 locus.
(EPS)

Figure S23 Forest plot and PM-plot for Chr11:114083173 locus.
(EPS)

Figure S24 Forest plot and PM-plot for Chr14:33632464 locus.
(EPS)

Figure S25 Forest plot and PM-plot for Chr15:21194226 locus.
(EPS)

Figure S26 Forest plot and PM-plot for Chr15:59860191 locus.
(EPS)

Figure S27 Forest plot and PM-plot for Chr17:46530712 locus.
(EPS)

Figure S28 Forest plot and PM-plot for Chr18:82240606 locus.
(EPS)

Figure S29 Forest plot and PM-plot for Chr19:3319089 locus.
(EPS)

Figure S30 Forest plot and PM-plot for ChrX:151384614 locus.
(EPS)

Table S1 False positive rate of RE versus traditional Wald Test based approaches at thresholds of increasing significance.
(EPS)

Table S2 False positive rate of HE versus traditional Wald Test based approaches at thresholds of increasing significance.
(EPS)

Text S1 Details on power and type I error simulations for gene by environment and 17 HDL mouse studies.
(PDF)
