# Supplementary material for: Meta-Analysis Identifies Gene-by-Environment Interactions as Demonstrated in a Study of 4,965 Mice
Source: PLoS Genet. 2014 Jan 9;10(1):e1004022. doi: 10.1371/journal.pgen.1004022 (PMC3886926; doi:10.1371/journal.pgen.1004022)
Supplement: Text S1 — Details on power and type I error simulations for gene by environment and 17 HDL mouse studies. (PDF) [file pgen.1004022.s033.pdf]

Meta-analysis identifies gene-by-environment interactions as demonstrated in a study of 4,965 mice

Eun Yong Kang*, Buhm Han*, Nicholas Furlotte*, Jong Wha Joo, Diana Shih, Richard C. Davis, Aldons J. Lusis**, Eleazar Eskin**

Contents

1 Power comparison between Meta-GxE, fixed effects meta-analysis and Heterogeneity testing approach **2**

2 Power and type I error comparison between Meta-GxE approach and traditional Wald test based GxE testing approach **2**

3 Details of 17 HDL mouse studies **3**

3.1 BxH-Apoe(M/F) 3

3.2 BxD-db-5(M/F) and BxD-db-12(M/F) 3

3.3 HMDP-chow(M) 4

3.4 HMDP-fat(M/F) 4

3.5 HMDPxB-chow(M/F) and HMDPxB-ath(M/F) 4

3.6 CxB-ldlr(M/F) 5

3.7 BxH-wt(M/F) 5

1 Power comparison between Meta-GxE, fixed effects meta-analysis and Heterogeneity testing approach

We estimate power for the case in which the genetic effect exists in a subset of the studies. In our simulation study, assuming five studies of equal sample size of 1,000, we estimate power when all five studies exhibited the effect, when only 4 studies exhibited the effect, and so on. Supplementary Figure S1 shows that as the number of studies with effect decreases, the power of FE drops. In contrast, the RE method maintains high power regardless of the number of studies exhibiting and effect. The power of HE method is increasing as there exists more heterogeneity due to gene-by-environment interactions. Note that RE method is more powerful than HE method when there are 2 studies having interaction effects. In this case, two studies have interaction effects and three studies do not have an effect. Since the magnitude and direction of effects are same in 2 studies with interaction effects, the regression for estimating effect sizes will behavior as if there exists a common effect with effect size $\frac{2}{5}\beta$. For this reason, RE method is most powerful over HE and FE in this case. We applied FE analysis method to 17 HDL studies. Supplementary Figure S2 shows the Manhattan plot of the result of FE analysis. The FE method found fewer (14) significant loci compared to the 26 discovered by Meta-GxE.

2 Power and type I error comparison between Meta-GxE approach and traditional Wald test based GxE testing approach

To compare the power and type I error (false positive rate) between the Meta-GxE approach and traditional Wald test based approach to identify gene-by-environment interactions, we first used each approach to identify both environment-shared and environment-specific effect on the phenotype in simulated data sets. For this power comparison, we generated 6 simulated genotype data sets with 1000 individuals assuming a minor allele frequency of 0.3 and we simulated the phenotype using equation (3). For all 6 simulated studies, we assume that there is no environment-shared effect ($\beta = 0$), We also assume that one study has no gene-by-environment effect ($\gamma_i = 0$) and some of the remaining 5 studies have gene-by-environment interactions ($\gamma_i \neq 0$). Supplementary Figure S1 and

S2 show the power comparison of RE approach vs traditional Wald Test based approach and that of HE approach vs traditional Wald Test based approach. The proposed RE and HE approaches consistently outperform the traditional Wald test based testing framework. Supplementary Table S1 and S2 show the false positive rate comparison of RE approach vs traditional Wald Test based approach and that of HE approach vs traditional Wald Test based approach. Both the RE and HE approaches correctly control the false positive rate.

3 Details of 17 HDL mouse studies

Here we provide details of each of the mouse genetic studies involved in the meta-analysis.

3.1 BxH-Apoe(M/F)

C57BL/6J Apoe$^{-/-}$ (B6.Apoe$^{-/-}$) were purchased from Jackson Laboratory (Bar Harbor, Maine, United States). C3H/HeJ Apoe$^{-/-}$ (C3H.Apoe$^{-/-}$) were generated by backcrossing B6.Apoe$^{-/-}$ to C3H for ten generations. F1 mice were generated from reciprocal intercrossing between B6.Apoe$^{-/-}$ and C3H.Apoe$^{-/-}$, and F2 mice were subsequently bred by intercrossing F1 mice. A total of 334 F2 mice (169 female, 165 male) were produced. All mice were fed Purina Chow containing 4% fat until 8 wk of age and then transferred to a Western diet containing 42% fat and 0.17% cholesterol for 16 wk. Mice were euthanized and plasma collected at 24 wk of age [7].

3.2 BxD-db-5(M/F) and BxD-db-12(M/F)

We previously carried out an F2 intercross between the inbred strains DBA/2 and C57BL/6 [2]. These parental mice were obtained from The Jackson Laboratory (Bar Harbor, ME). The male C57BL/6 parents carried heterozygosity deficiency in the leptin receptor (db +/-) and F1 progeny were selected for homozygosity of the mutant allele. Among F2 progeny, only those with homozygous deficiency in leptin receptor (db/db) were selected. Animals were fed a chow diet with 6% fat by weight. Plasma levels of HDL were measured in both genders at 5 weeks of age (BxD-db-5(M/F)) and at 12 weeks of age (BxD-db-12(M/F)).

3.3 HMDP-chow(M)

Male mice from the hybrid HMDP panel were purchased from The Jackson Laboratory and studied as previously described [1]. Mice were between 6 and 10 wk of age on arrival and to ensure adequate acclimatization to a common environment the mice were aged until 16 wk of age. All mice were maintained on a chow diet (Ralston-Purina Co.) until sacrifice at 16 wk of age. Following a 16-h fast, mice were bled retro-orbitally under isoflurane anesthesia.

3.4 HMDP-fat(M/F)

All mice were obtained from The Jackson Laboratory and were bred at University of California, Los Angeles to generate mice used in this study as previously described [4]. Purchased mice were maintained on a chow diet (Ralston Purina Company) until 8 weeks of age. Then they were given a high-fat, high-sucrose diet (Research DietsD12266B) with the following composition: 16.8% kcal protein, 51.4% kcal carbohydrate, and 31.8% kcal fat for 8 weeks. Plasma was collected at 16 weeks of age.

3.5 HMDPxB-chow(M/F) and HMDPxB-ath(M/F)

Mice for the HMDPxB panel were created by breeding females of the various HMDP inbred strains [1] to males carrying transgenes for both Apoe Leiden [5] and for human Cholesterol Ester Transfer Protein (CETP) [3] on a C57BL/6 genetic background. Male and female F1 progeny were genotyped to verify presence of both transgenes and then provided a normal chow diet until 8 weeks of age. At that time, after a 4h fast, animals were anesthetized with isoflurane vapor and a sample of blood (HMDPxB-chow) was collected via the retro orbital sinus and plasma was prepared using EDTA coated tubes (Cat.365973, Becton Dickinson, Franklin Lakes, NJ) . These plasmas represent the HMDPxB-chow(M) (male) and HMDPxB-chow(F) (female) populations. The animals were then fed a high-fat, high-cholesterol diet (33 kcal% fat (mostly cocoa butter) + 1% cholesterol) (Research Diets, New Brunswick, NJ) for 16 weeks at which time a second blood sample was collected by the same procedures. These plasmas represent the HMDPxB-ath(M) (male) and HMDPxB-ath(F) (female) populations. All mice were maintained on a 12h light and 12h dark cycle and fed ad

libitum.

3.6 CxB-ldlr(M/F)

Female BALB/cByJ-LDLRKO (designated as C) mice were crossed with male C57BL/6J-LDLRKO (designated as B) to generate F1 mice. Intercross of F1 was performed to generate F2 mice. A total of 124 male and 64 female F2 mice were phenotyped and genotyped. These F2 mice were fed a Western diet (Harlan Teklad) for 12 weeks before euthanasia and collection of plasma. For Genotyping, Illumina 1440 SNP Mouse medium density microarray was used with 799 informative SNPs. Two additional SNPs at 30 Mb and 67 Mb of chromosome 2 were also typed manually. Therefore a total of 801 markers were used. The average marker density was 1 SNP per 2.2 Mb (Unpublished).

3.7 BxH-wt(M/F)

BXH wild type (BXH/wt) mice were produced as previously described [6]. Briefly, C57BL/6J mice were intercrossed with C3H/HeJ mice to generate 321 F2 progeny (161 females, 160 males). These mice were fed Purina Chow (Ralston-Purina) containing 4% fat until 8 wk of age, after which, they were fed a western diet (Teklad 88137, Harlan Teklad) containing 42% fat and 0.17% cholesterol. At 20 weeks of age, plasma samples were collected as described above. All mice were maintained on a 12 h light and 12 h dark cycle and fed ad libitum.

References

[1] Brian J. Bennett, Charles R. Farber, Luz Orozco, Hyun Min Kang, Anatole Ghazalpour, Nathan Siemers, Michael Neubauer, Isaac Neuhaus, Roumyana Yordanova, Bo Guan, Amy Truong, Wen-pin P. Yang, Aiqing He, Paul Kayne, Peter Gargalovic, Todd Kirchgessner, Calvin Pan, Lawrence W. Castellani, Emrah Kostem, Nicholas Furlotte, Thomas A. Drake, Eleazar Eskin, and Aldons J. Lusis. A high-resolution association mapping panel for the dissection of complex traits in mice. *Genome Res*, 20(2):281–90, 2 2010.

[2] Richard C. Davis, Atila van Nas, Lawrence W. Castellani, Yi Zhao, Zhiqiang Zhou, Pingzi Wen, Suzanne Yu, Hongxiu Qi, Melenie Rosales, Eric E. Schadt, Karl W. Broman, Miklós Péterfy, and Aldons J. Lusis. Systems genetics of susceptibility to obesity-induced diabetes in mice. *Physiol Genomics*, 44(1):1–13, 1 2012.

[3] X. C. Jiang, L. B. Agellon, A. Walsh, J. L. Breslow, and A. Tall. Dietary cholesterol increases transcription of the human cholesteryl ester transfer protein gene in transgenic mice. dependence on natural flanking sequences. *J Clin Invest*, 90(4):1290–5, 10 1992.

[4] Brian W. Parks, Elizabeth Nam, Elin Org, Emrah Kostem, Frode Norheim, Simon T. Hui, Calvin Pan, Mete Civelek, Christoph D. Rau, Brian J. Bennett, Margarete Mehrabian, Luke K. Ursell, Aiqing He, Lawrence W. Castellani, Bradley Zinker, Mark Kirby, Thomas A. Drake, Christian A. Drevon, Rob Knight, Peter Gargalovic, Todd Kirchgessner, Eleazar Eskin, and Aldons J. Lusis. Genetic control of obesity and gut microbiota composition in response to high-fat, high-sucrose diet in mice. *Cell Metab*, 17(1):141–52, 1 2013.

[5] A. M. van den Maagdenberg, M. H. Hofker, P. J. Krimpenfort, I. de Bruijn, B. van Vlijmen, H. van der Boom, L. M. Havekes, and R. R. Frants. Transgenic mice carrying the apolipoprotein e3-leiden gene exhibit hyperlipoproteinemia. *J Biol Chem*, 268(14):10540–5, 5 1993.

[6] Atila van Nas, Leslie Ingram-Drake, Janet S. Sinsheimer, Susanna S. Wang, Eric E. Schadt, Thomas Drake, and Aldons J. Lusis. Expression quantitative trait loci: replication, tissue- and sex-specificity in mice. *Genetics*, 185(3):1059–68, 7 2010.

[7] S. Wang, N. Yehya, E.E. Schadt, H. Wang, T.A. Drake, and A.J. Lusis. Genetic and genomic analysis of a fat mass trait with complex inheritance reveals marked sex specificity. *PLoS genetics*, 2(2):e15, 2006.
